# Mitotic slippage and the subsequent cell fates after inhibition of Aurora B during tubulin-binding agent–induced mitotic arrest

**DOI:** 10.1038/s41598-017-17002-z

**Published:** 2017-12-01

**Authors:** Yasuo Tsuda, Makoto Iimori, Yuichiro Nakashima, Ryota Nakanishi, Koji Ando, Kippei Ohgaki, Hiroyuki Kitao, Hiroshi Saeki, Eiji Oki, Yoshihiko Maehara

**Affiliations:** 1 Departments of Surgery and Science, Graduate School of Medical Sciences, Kyushu University, 3-1-1 Maidashi, Higashi-ku, Fukuoka, 812-8582 Japan; 2 Department of Molecular Cancer Biology, Graduate school of Pharmaceutical Sciences, Kyushu University, 3-1-1 Maidashi, Higashi-ku, Fukuoka, 812-8582 Japan

## Abstract

Tubulin-binding agents (TBAs) are designed to target microtubule (MT) dynamics, resulting in compromised mitotic spindles and an unsatisfied spindle assembly checkpoint. The activity of Aurora B kinase is indispensable for TBA-induced mitotic arrest, and its inhibition causes mitotic slippage and postmitotic endoreduplication. However, the precise phenomenon underlying mitotic slippage, which is caused by treatment with both Aurora B inhibitors and TBAs, and the cell fate after postmitotic slippage are not completely understood. Here, we found that HeLa and breast cancer cells treated with the different types of TBAs, such as paclitaxel and eribulin (MT-stabilizing and MT-destabilizing agents, respectively), exhibited distinct behaviors of mitotic slippage on inhibition of Aurora B. In such conditions, the cell fates after postmitotic slippage vastly differed with respect to cell morphology, cell proliferation, and cytotoxicity in short-term culture; that is, the effects of inhibition of Aurora B were beneficial for cytotoxicity enhancement in eribulin treatment but not in paclitaxel. However, in long-term culture, the cells that survived after mitotic slippage underwent endoreduplication and became giant cells in both cases, resulting in cellular senescence. We propose that MT-destabilizing agents may be more appropriate than MT-stabilizing agents for treating cancer cells with a weakened Aurora B kinase activity.

## Introduction

Microtubules (MTs) are highly dynamic polymers that constantly switch between phases of growth and shrinkage^1–3^. In mitotic cells, MTs constitute the spindle, and their plus-end dynamics is required for capture of kinetochores and equal segregation of sister chromatids, which are essential for proper mitotic progression. Disruption of spindle MTs does not satisfy the spindle assembly checkpoint (SAC), which causes induction of mitotic arrest, thereby leading to cell death^4^. Many chemical compounds can bind to MTs and induce SAC-dependent mitotic arrest; such tubulin-binding agents (TBAs) serve as important chemotherapeutic drugs against tumor cells^5,6^.

TBAs are mainly classified into MT-stabilizing and MT-destabilizing agents. The former is exemplified by taxans and epothilones, and the latter, by vinca alkaloids, vinorelbine, and eribulin, which is a synthetic analogue of halichondrin B, is the newest anti-tumor drug for breast cancer by inhibiting MT polymerization irreversibly. Although both kinds of TBAs suppress MT dynamics and induce mitotic arrest identically, these agents affect MT dynamics in completely different ways. MT-stabilizing agents, such as taxans, directly bind along the interior surface of the MTs with high affinity, but bind poorly to soluble tubulin, resulting in inhibition of MT dynamics^7^. MT-destabilizing agents, such as vinca alkaloids and eribulin, bind to the β-subunit of tubulin dimers at the MT plus-ends with high affinity, suppressing MT dynamics^8–11^. However, eribulin binds to MT plus-ends with high affinity in a concentration-independent manner, whereas vinca alkaloids bind not only to MT plus-ends but also to tubulin located along the sides of MTs at a high concentration, suggesting that eribulin and vinca alkaloids inhibit microtubule dynamics via different mechanisms^11–14^. Cancer cells treated with both kinds of TBAs exhibit distinct cell fates following prolonged exposure to MT-stabilizing (e.g., paclitaxel) or MT-destabilizing (e.g., nocodazole) agents^4,15,16^. TBAs activate the SAC, leading to mitotic arrest; Aurora B kinase activity is required to maintain SAC signaling, as its inhibition prevents recruitment of all SAC components to kinetochores^17^.

Aurora B is a member of the Aurora kinase family, which comprises three family members, that is, Aurora A, B, and C. Aurora B is a component of chromosomal passenger complex (CPC), which consists of kinase and three nonenzymatic subunits, that is, INCENP, survivin, and borealin, which regulate the localization, enzymatic activity, and stability, respectively, of Aurora B kinase^18^. In early mitosis, many kinetochores engage in incorrect MT attachments. To ensure equal chromosome segregation, Aurora B kinase engages in kinetochore–MT error correction, particularly in relation to destabilization of kinetochore–MT interactions. Aurora B kinase activity is required to maintain the SAC induced by paclitaxel; inhibition of Aurora B rapidly overrides mitotic arrest (hereafter referred to as “mitotic slippage”)^19–21^. Several studies have reported that mitotic slippage is caused by treatment with both paclitaxel (MT-stabilizing agent) and Aurora B inhibitors. However, the cell fate after postmitotic slippage is not completely clear. Furthermore, in the case of treatment with both eribulin (MT-destabilizing agent) and Aurora B inhibitors, the cell fate also remains poorly understood.

Here, we investigated the contribution of Aurora B activity to maintaining the SAC induced by paclitaxel, an MT-stabilizing agent, or eribulin, an MT-destabilizing agent. We also investigated the cell fate after postmitotic slippage, including cell morphology, cell proliferation, cytotoxicity, and cellular senescence. Importantly, in breast cancer, it has been reported that expression of Aurora B is heterogeneous and is not correlated with clinicopathological factors or prognosis^22^. A comparison of mitotic cell response, including mitotic slippage, and postmitotic cell fates following paclitaxel and eribulin treatment when Aurora B is inhibited may help to select drugs for the clinical treatment of breast cancer.

## Results

### Paclitaxel and eribulin induced mitotic arrest and Aurora B activation

**Figure 1:**
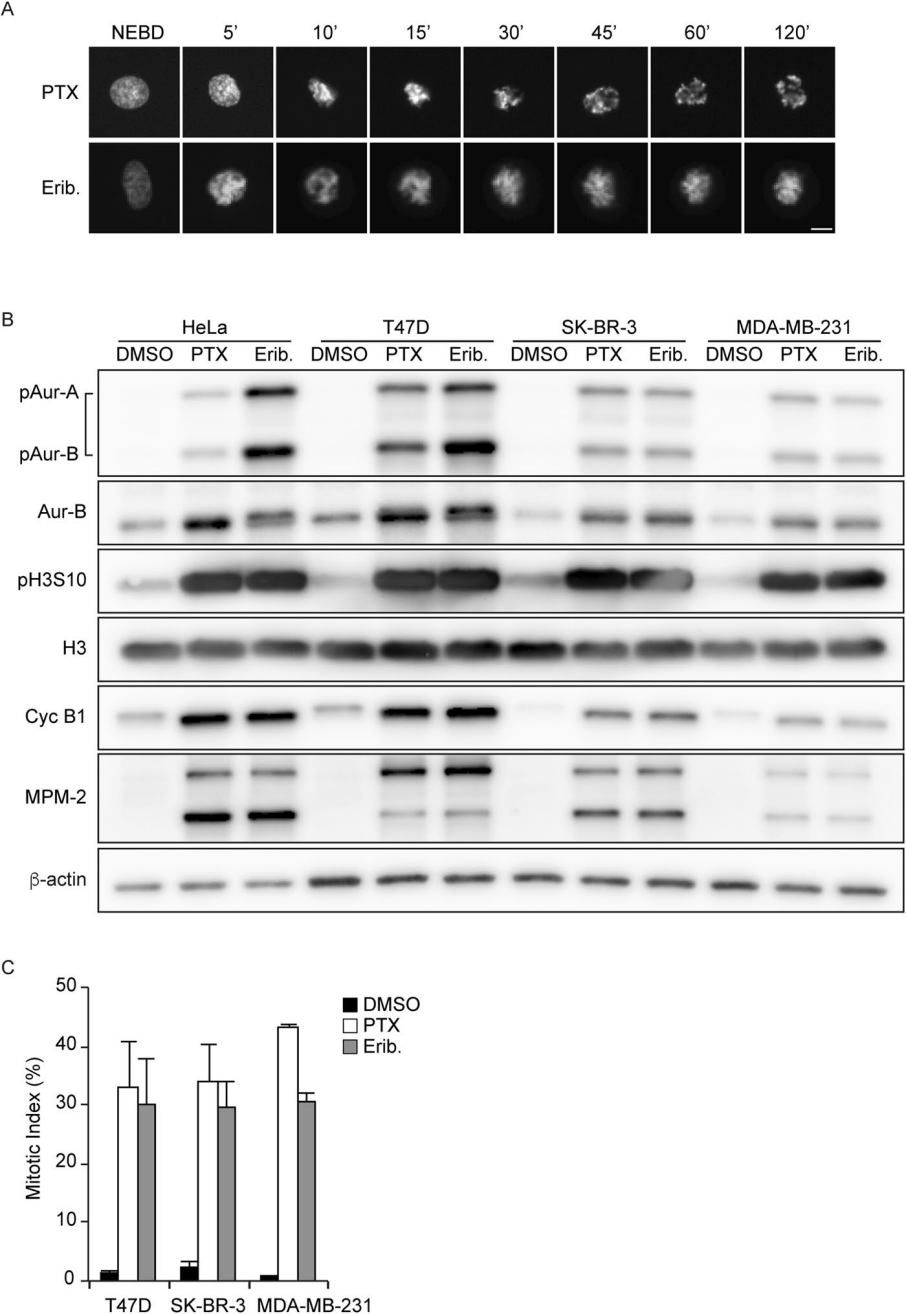
Effect of paclitaxel and eribulin on mitosis. (**A**) Select frames from live-cell imaging of HeLa cells expressing histone H2B-GFP. Synchronized HeLa cells were cultured with 100 nM paclitaxel (PTX) or 10 nM eribulin (Erib). Time (min) after nuclear envelope breakdown (NEBD) is shown on the images. Scale bars, 5 µm. (**B**) Synchronized HeLa cells were cultured with DMSO, 100 nM PTX, or 10 nM Erib for 16 h. Immunoblot analysis was performed using antibodies against the indicated proteins. T47D, SK-BR-3, and MDA-MB-231 cells were cultured with DMSO, 100 nM PTX, or 10 nM Erib for 16 h. Immunoblot analysis was performed using antibodies against the indicated proteins. (**C**) T47D, SK-BR-3, and MDA-MB-231 cells were cultured with DMSO, 100 nM PTX, or 10 nM Erib for 16 h. The mitotic index was determined by analyzing MPM2-positive cells. Data are mean values from three independent experiments; error bars, ± SD, number of cells, >10,000 per experiment.

To determine the effect of paclitaxel and eribulin on mitosis, we examined live-cell imaging in HeLa cells stably expressing GFP-tagged histone H2B (hereafter referred to as HeLa H2B-GFP cells)^23^. Mitosis of HeLa cells was arrested on treatment with paclitaxel or eribulin, used at 100 nM and 10 nM, respectively, which are the minimum concentrations required for mitotic arrest (Supplementary Fig. 1), and chromosome segregation was inhibited by suppression of MT dynamics (Fig. 1A). Consistent with these observations, immunoblotting analysis revealed that treatment with paclitaxel or eribulin induced the accumulation of mitotic marker proteins, such as cyclin B1, MPM-2, and phospho-histone H3 Ser10 (Fig. 1B). Furthermore, the observed mitotic arrest involved simultaneous activating phosphorylation of Aurora B at Thr-232 and of Aurora A at Thr-288 (Fig. 1B).

Paclitaxel and eribulin are currently used as clinically important chemotherapy drugs for breast cancer^24,25^. Breast cancer is divided into several subtypes according to the estrogen receptor (ER), progesterone receptor (PgR), and HER2 status, which are indicators for therapeutic strategy. To examine whether paclitaxel and eribulin induce mitotic arrest in breast cancer cell lines, we selected typical subtypes of breast cancer cell lines, such as T47D (ER and PgR positive), SK-BR-3 (Her2 positive), and MDA-MB-231 (triple negative), and confirmed that both paclitaxel and eribulin induced mitotic arrest in all the breast cancer cell lines used, regardless of subtypes (Fig. 1B,C). Thus, we conclude that both paclitaxel and eribulin induced mitotic arrest and increased Aurora B activity in HeLa cells and among different subtypes of breast cancer cell lines.

### Inhibition of Aurora B rapidly led to premature exit from mitotic arrest in paclitaxel-treated cells but this exit was not rapid in eribulin-treated cells

**Figure 2:**
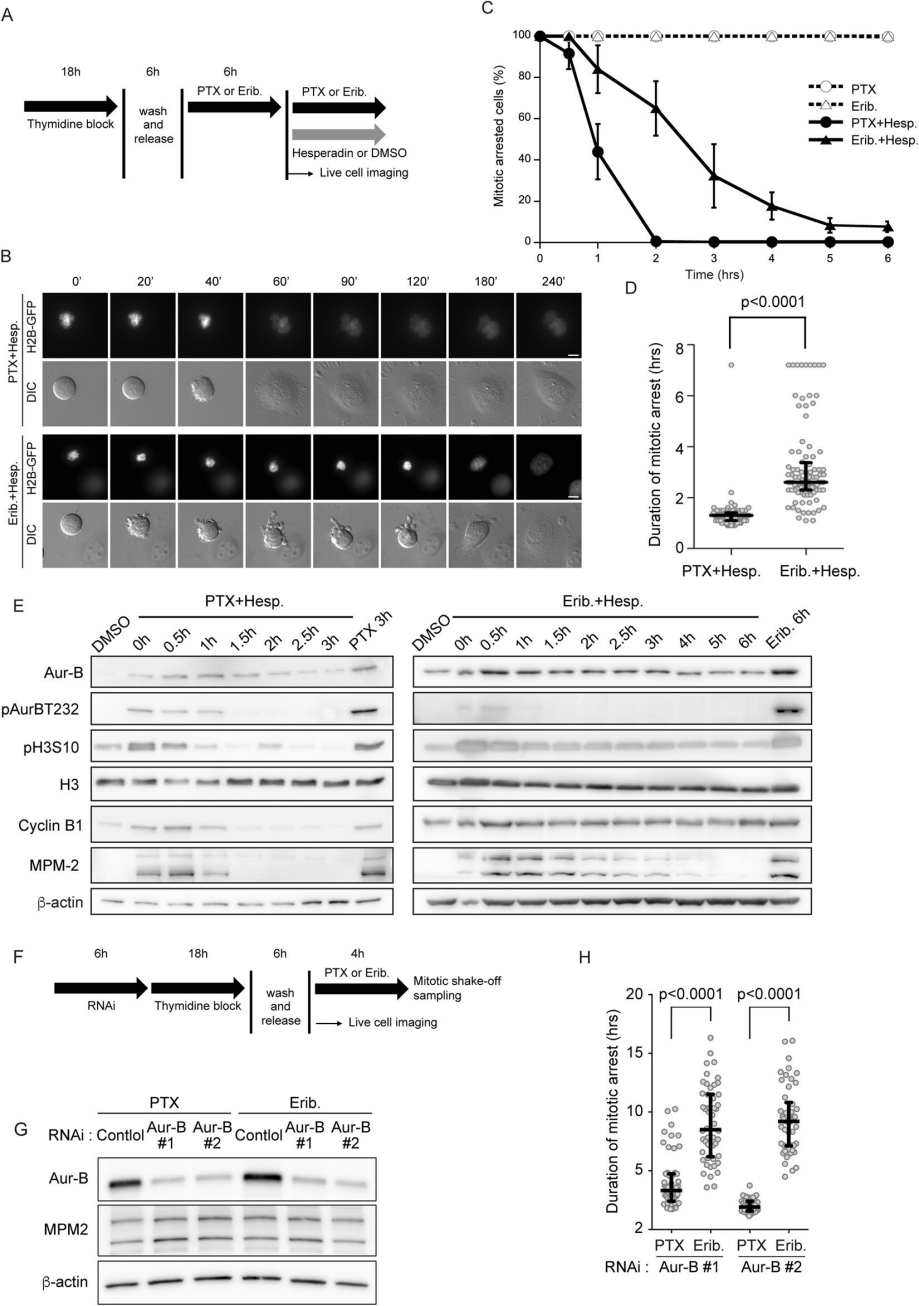
Effect of inhibition of Aurora B during paclitaxel- or eribulin-induced mitotic arrest. (**A**) Schemes of experiments shown in Fig. 2B–D. (**B**) Select frames from live-cell imaging of representative HeLa/H2B-GFP cells. Synchronized HeLa/H2B-GFP cells were cultured with 100 nM paclitaxel (PTX) or 10 nM eribulin (Erib) for 6 h after release. Then, DMSO or 50 µM hesperadin (Hesp) was added. Time (min) after the addition of hesperadin or DMSO is shown on the images. Scale bar, 5 µm. (**C**) Percentage of cells arrested in mitosis in the presence of the different drugs. Live-cell images were analyzed at 6 h from the addition of Hesp or DMSO. Data are mean values from three independent experiments; error bars, ± SD, number of cells, 100 per experiment. (**D**) Live-cell images were analyzed from the time of addition of Hesp to the onset of mitotic exit in PTX + Hesp and Erib + Hesp cells. Open circles represent individual cells. The duration of mitotic arrest was measured from the time of addition of hesperadin to the onset of mitotic exit in every 100 cells per experiment. Horizontal bar, median; vertical bar, interquartile range. (**E**) Synchronized HeLa cells were cultured with 100 nM PTX or 10 nM Erib for 6 h. HeLa cells that underwent mitosis arrest were harvested by mitotic shake-off and transferred into fresh medium containing the test drugs. Immunoblot analysis was performed using antibodies against the indicated proteins. The levels of MPM-2 and cyclin B1 were determined as the mitosis-specific markers. (**F**) Schemes of experiments shown in Fig. 2G-H. (**G**) HeLa cells were transfected with Aurora B siRNAs as indicated; after then, cells were synchronized by a single thymidine block and then after 6 h released into PTX or Erib for 4 h. Mitotic cells were harvested by mitotic shake-off. Immunoblot analysis was performed using antibodies against the indicated proteins. The levels of MPM-2 were determined as a mitosis-specific marker. (**H**) Aurora B depleted- and synchronized-HeLa cells were prepared as shown in F-G. Live-cell images were analyzed from the time of NEBD to chromosome decondensation in PTX- or Erib-treated cells (n ≥ 50) from three independent experiments. Open circles represent individual cells. Horizontal bar, median; vertical bar, interquartile range.

Previous studies showed that inhibition of Aurora B rapidly overrides paclitaxel-induced mitotic arrest^19–21^. However, the cell behavior on inhibition of Aurora B activity during eribulin-induced mitotic arrest is not known. We treated HeLa H2B-GFP cells with paclitaxel or eribulin and then added hesperadin, an Aurora B kinase inhibitor^20^; then, we followed the cells over time by live cell imaging. Consistent with a previous study^20^, we found that almost all of the paclitaxel-treated cells exited mitosis within 2 h after addition of hesperadin. However, although eribulin-treated cells also exited mitosis after addition of hesperadin, they remained in mitosis longer than cells treated with paclitaxel and hesperadin (Fig. 2A–C). Moreover, we measured the duration from hesperadin addition to chromosome decondensation. In contrast to the cells treated with paclitaxel and hesperadin, those treated with eribulin and hesperadin varied significantly in length and homogeneity of duration of mitotic arrest (Fig. 2D). Furthermore, we monitored the cyclin B1 and MPM-2 levels by immunoblotting. After hesperadin addition, the cyclin B1 and MPM-2 levels decreased rapidly in paclitaxel-treated HeLa cells. In contrast, in eribulin-treated HeLa cells, the levels of these markers decreased more slowly than those in paclitaxel-treated cells (Fig. 2E). To confirm these observations, we further analyzed the effect of Aurora B inhibition during mitotic arrest induced with paclitaxel or eribulin by using barasertib, a highly selective Aurora B inhibitor. We first determined the optimal concentration of barasertib to specifically inhibit Aurora B. HeLa cells were treated with five concentrations of barasertib during mitotic arrest induced by nocodazole. The specificity of Aurora B inhibition was confirmed by the disappearance of Aurora B phosphorylated at Thr-232, but not of Aurora A phosphorylated at Thr-288. Consistent with a previous study^26^, 1 µM barasertib specifically inhibited Aurora B (Supplementary Fig. 2A). There was a time-dependent decrease in mitotic rounding cells upon treatment with paclitaxel and barasertib, but not upon treatment with eribulin and barasertib (Supplementary Fig. 2B and C). The levels of cyclin B1 and MPM-2 decreased rapidly in cells treated with paclitaxel and barasertib. By contrast, the levels of these mitotic markers were not obviously decreased in cells treated with eribulin and barasertib (Supplementary Fig. 2D). Consistent with these observations obtained using Aurora B inhibitors, eribulin-treated cells remained in mitosis longer than paclitaxel-treated cells upon Aurora B knockdown via RNAi (Fig. 2F–H). Collectively, these results suggest that inhibition of Aurora B activity during mitotic arrest induced with paclitaxel or eribulin led to mitotic slippage and that the manner of slippage differed completely for both TBAs.

### Eribulin was affected less by inhibition of Aurora B in breast cancer cell lines

Both paclitaxel and eribulin induce mitotic arrest and increase Aurora B activity among different subtypes of breast cancer cell lines (Fig. 1B,C). Therefore, we further examined the effect of inhibition of Aurora B during mitotic arrest induced with paclitaxel or eribulin among different subtypes of breast cancer cell lines. We used ZM447439 or hesperadin as an Aurora B inhibitor. Cells were treated with 100 nM paclitaxel or 10 nM eribulin, except for MDA-MB-231 cells, which were treated with 100 nM eribulin, all of which are minimum concentrations required for mitosis arrest of breast cancer cell lines (Supplementary Fig. 1). Asynchronous T47D cells were cultured in media supplemented with paclitaxel or eribulin and Aurora B inhibitors, resulting in decrease in MPM-2 and cyclin B1 levels. Furthermore, MPM-2 and cyclin B1 levels on treatment with paclitaxel and Aurora B inhibitors were remarkably lower than those with eribulin and Aurora B inhibitors (Supplementary Fig. 3A). Consistent with this result, the mitotic index of paclitaxel and Aurora B inhibitors greatly decreased by approximately one-sixth. In contrast, the mitotic index of eribulin and Aurora B inhibitors decreased only by approximately one-third (Supplementary Fig. 3B). We also studied SK-BR-3 and MDA-MB-231 cells, which differ from T47D cells; the same trend was observed in both cell lines (Supplementary Fig. 3C–F). These results suggest that the dependency of Aurora B kinase activity on eribulin-induced mitotic arrest was lesser than that for paclitaxel and that this phenomenon was conserved among different subtypes of breast cancer cell lines.

### Aurora B inhibition–induced mitotic slippage resulted in aberrant nuclear structure and postmitotic endoreduplication

**Figure 3:**
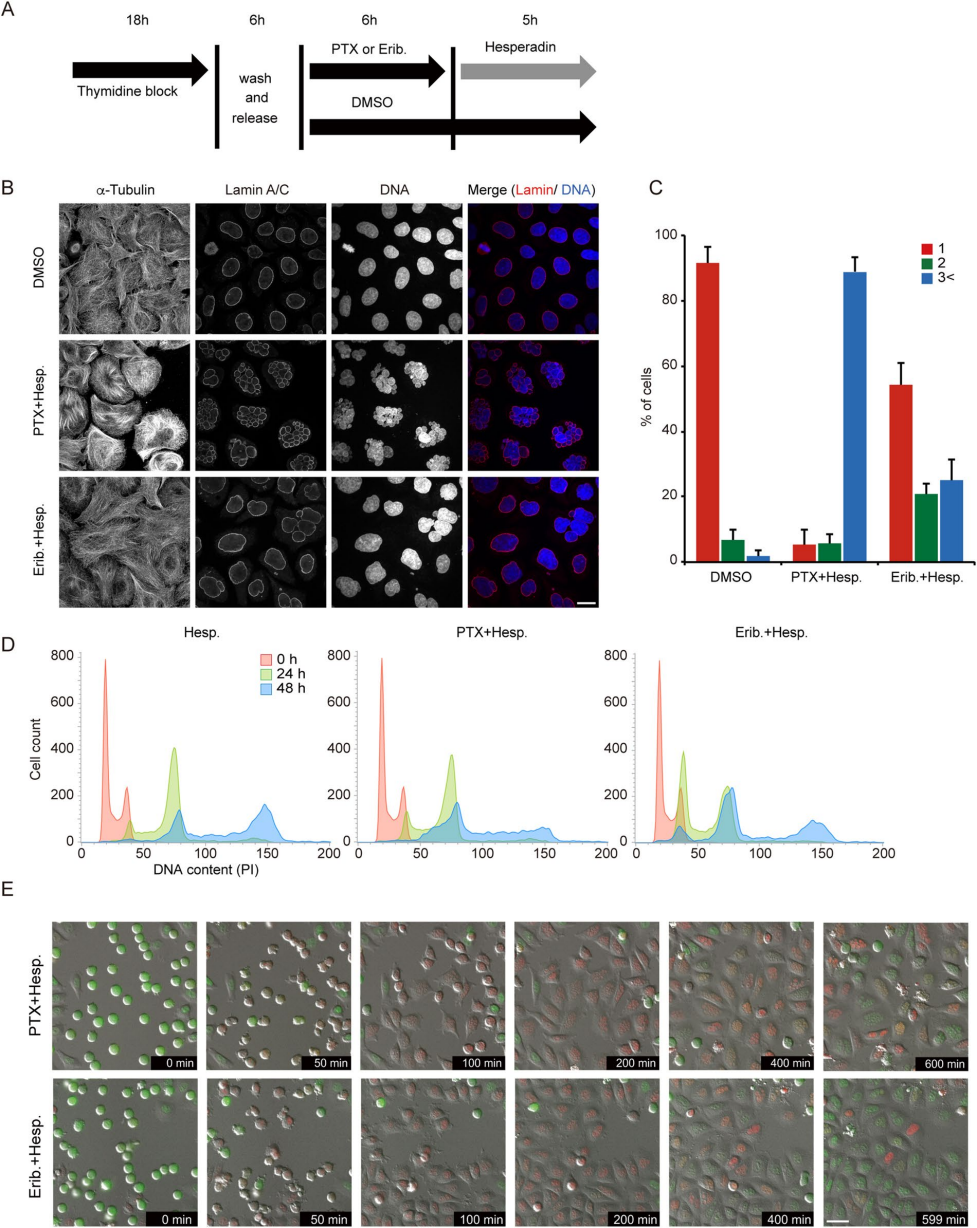
Cell morphology and cell cycle progression in postmitotic slippage. (**A**) Schemes of experiments shown in Fig. 3B,C. (**B**) Representative confocal images showing the morphology of nuclei in postmitotic slippage HeLa cells. Cells were costained with antibodies against tubulin, lamin A/C (red), and DAPI (blue). Quantification of the proportion of nuclei in postmitotic slippage cells is shown in (**C)**. Data are mean values from three independent experiments; error bars, ± SD, number of cells, >100 per experiment. Scale bar, 10 µm. (**D**) Flow cytometry cell cycle analysis using propidium iodide (PI) DNA staining. Asynchronous HeLa cells were treated with the indicated drugs. (**E**) Select frames from live-cell imaging of HeLa-Fucci cells expressing both mCherry-Cdt1 and mVenus-Geminin. See Fig. 2A for the experimental scheme. Scale bar, 50 µm.

Inhibition of Aurora B led to mitotic slippage from TBA-induced mitotic arrest; however, the manner of slippage differed between paclitaxel and eribulin. We further investigated the phenotypes of postmitotic slippage, which was induced by inhibition of Aurora B under the status of TBA-induced mitotic arrest. A previous study reported that inhibition of Aurora B rapidly overrides the mitotic arrest induced by paclitaxel, resulting in enlarged cells with multiplenuclei and micronuclei^20^. Consistent with that study, multiplenuclei and micronuclei were observed just after mitotic slippage induced by treatment with paclitaxel and hesperadin. In contrast, our data showed that a single nucleus was present in approximately half of the cells treated with eribulin and hesperadin (Fig. 3A–C).

We next examined cell-cycle status after mitotic slippage. Polyploidy is induced in cells treated with Aurora B-targeting siRNA or Aurora B inhibitors, including hesperadin^27–29^. Our observations in cells treated with hesperadin alone or together with paclitaxel were consistent with these studies, and cells treated with hesperadin and eribulin also exhibited similar phenotypes (Fig. 3D). To visualize the cell-cycle status, we performed live-cell imaging of HeLa-Fucci (fluorescent ubiquitination–based cell-cycle indicator) cells expressing both mCherry-Cdt1 and mVenus-Geminin^30,31^. After mitotic slippage, the aberrant nuclear structures noted for treatment with paclitaxel and hesperadin differed from those for treatment with eribulin and hesperidin. However, in both cases, transition from the G_1_ phase (red nuclei) to the S phase (green nuclei) was noted (Fig. 3E). Then, the cells underwent re-entry to the next mitosis and mitotic slippage occurred again (Supplementary Fig. 4). Taken together, these data suggest that phenotypes of morphological changes in nuclei and postmitotic endoreduplication are caused by Aurora B inhibitor–induced mitotic slippage not only in the case of paclitaxel-induced mitotic arrest but also in eribulin-induced mitotic arrest.

### Inhibition of Aurora B enhanced the cytotoxicity of eribulin but suppressed the cytotoxicity of paclitaxel

**Figure 4:**
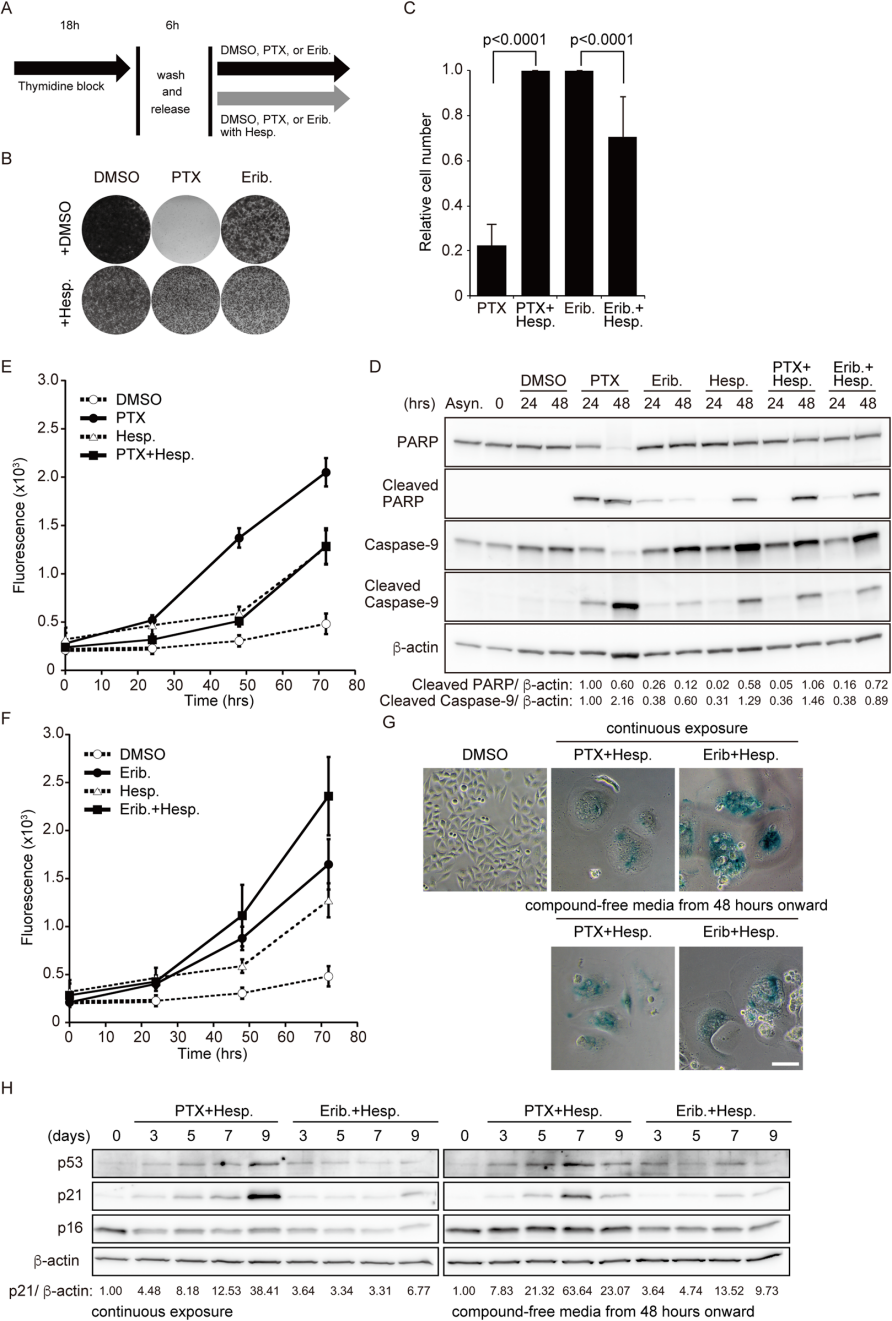
Cell proliferation, cytotoxicity, and cellular senescence in postmitotic slippage. (**A**) Schemes of experiments shown in Fig. 4B–H. (**B**,**C**) Synchronized HeLa cells were treated with DMSO, 100 nM paclitaxel (PTX), or 10 nM eribulin (Erib) alone or in combination with 50 µM hesperadin (Hesp) for 72 h. Representative images of colony formation by crystal violet staining are shown in B. Relative cell number were quantified in (**C)**. Data are mean values from three independent experiments. (**D**) Immunoblot analysis was performed using antibodies against the indicated proteins and is shown in **D**. The levels of cleaved PARP and cleaved caspase-9, which are apoptosis-specific markers, were determined. Levels of cleaved PARP and cleaved caspase-9 protein, normalized against β-actin, are shown relative to the PTX treatment for 24 h (defined as 1). (**E**,**F**) Cell viability was analyzed using the CellTox Green Cytotoxicity Assay in the treatment of indicated drugs. The data in **E** and **F** were performed in the same lot of experiment. The plots of PTX and Erib were divided into **E** and **F**, respectively, in order to avoid confusion. Data are mean values from three independent experiments. (**G**) Representative images showing SA-β-gal activity in postmitotic slippage cells (7 days). (**H**) Immunoblot analysis was performed using antibodies against the indicated proteins. Levels of p21 protein, normalized against β-actin, are shown relative to control (defined as 1).

To verify the changes in the antitumor effect of paclitaxel and eribulin due to inhibition of Aurora B, we observed cell fates after mitotic slippage in HeLa cells. Previous studies showed that, with paclitaxel, most cells exited a prolonged mitotic arrest and then died in the following interphase without postmitotic endoreduplication^15,32^, whereas, with nocodazole, approximately half of the cells also exited from mitosis and then underwent endoreduplication, entering the second and third mitoses without ever undergoing division^15^. Consistent with these studies, the colony-forming efficiency greatly decreased after 72-h incubation with paclitaxel alone, whereas the efficiency with eribulin alone partially decreased (Fig. 4A–C). Here, we found that the colony-forming efficiency in cells treated with paclitaxel and hesperidin was markedly higher than that for paclitaxel alone. In contrast, treatment with eribulin-hesperadin was more sensitive to the colony formation than that of eribulin alone (Fig. 4A–C). Therefore, we attributed this difference to the induction level of apoptosis and measured active caspase and cleaved PARP levels to evaluate the induction of apoptosis. Both caspase-9 and cleaved PARP levels were higher in cells treated with paclitaxel alone than in those treated with paclitaxel and hesperidin. However, we observed an inverse tendency in cells treated with eribulin, that is, apoptosis levels in cells treated with eribulin and hesperidin were higher than those with eribulin alone (Fig. 4D).

To confirm these data, we assessed the time course of cell death. Consistent with our observations of apoptosis, the cytotoxic effect noted for cells treated with eribulin and hesperadin was clearly higher than that with eribulin alone (Fig. 4F), whereas the cells treated with paclitaxel and hesperadin had greater drug resistance than those treated with paclitaxel alone (Fig. 4E). We also examined this in T47D, SK-BR-3, and MDA-MB-231 cells and confirmed the presence of the same tendencies (Supplementary Fig. 5). Thus, we conclude that the cytostatic effect of eribulin is more effective in combination with an Aurora B inhibitor, but that of paclitaxel is adversely affected by combination with an Aurora B inhibitor.

### Mitotic slippage–induced endoreduplication triggered cellular senescence

Previous studies showed that chemotherapeutic agents could induce senescence in tumor cells, especially in the antiapoptotic context^33,34^. Furthermore, specific inhibition of Aurora B leads to aberrant mitotic exit and cellular senescence^20,21,27^. As our data showed that treatment with an Aurora B inhibitor induced mitotic slippage from both paclitaxel-induced and eribulin-induced mitotic arrest, resulting in postmitotic endoreduplication, we speculated that the cells in such a context might cause cellular senescence after long-term culture. To investigate this possibility, we measured SA-β-gal activity, a hallmark of cellular senescence^35^. Cells surviving for 5–7 days after treatment with paclitaxel and hesperadin or with eribulin and hesperadin showed an enlarged cellular morphology with increased SA-β-gal activity (Fig. 4G). A recent study reported that the p53–p21 pathway was upregulated under long-term arrest induced by specific inhibition of Aurora B in HeLa cells^27^. Although wild-type endogenous p53 is suppressed by human papillomavirus (HPV)–generated E6 activity in HeLa cells, it has been shown that p53 can escape from its E6-mediated downregulation after chemotherapy with drugs such as cisplatin^29^. Consistent with these observations, the p53–p21 pathway was found to be remarkably upregulated after long-term culture (days 7–9) not only in medium containing compounds (paclitaxel and hesperadin or eribulin and hesperadin) but also compound-free medium (Fig. 4H). Our results suggest that cells surviving after mitotic slippage and following long-term culture undergo morphological changes and cellular senescence.

## Discussion

TBAs are clinically important chemotherapeutic drugs against tumor cells. TBAs disrupt the dynamics of spindle MTs and activate the SAC, which causes induction of mitotic arrest, resulting in cell death^4^. However, mitotic cell death is not always induced in TBA-treated cells; previous studies demonstrated that TBA-induced cell death occurs not only during mitosis but also during interphase following mitotic slippage^15,32^. Furthermore, such TBA-induced mitotic arrest is maintained by Aurora B kinase in an activity-dependent manner^20,32^. Here, we found that Aurora B contributed to different extents to paclitaxel- and eribulin-induced mitotic arrest. We also focused on the distinct cell fates after postmitotic slippage induced by inhibition of Aurora B in the case of paclitaxel- and eribulin-induced mitotic arrest.

Aurora B plays multiple roles during cell division, including the processes of spindle assembly, kinetochore–MT interactions, and cytokinesis, and is believed to be involved in the maintenance of SAC signaling^17^. Consistent with previous studies, our data showed that inhibition of Aurora B during mitotic arrest induced with TBAs led to mitotic slippage. In addition, our data showed differing contributions of Aurora B to maintaining SAC induced-mitotic arrest in treatment with paclitaxel or eribulin. In previous studies, during nocodazole-induced mitotic arrest, Aurora B was found to be required for maintenance of SAC signaling, especially in retention of kinetochore localization of the checkpoint kinases BubR1 and Bub1, whereas Mad2 still localized at kinetochores after inhibition of Aurora B by hesperadin^15,20^. In the case of paclitaxel-induced mitotic arrest, cells with inhibition of Aurora B show marked decrease not only in BubR1- and Bub1- but also in Mad2-positive kinetochores, resulting in accelerated SAC satisfaction and mitotic slippage^20,21,36^. Taken together with our result showing that cells treated with eribulin and hesperadin remained in mitosis substantially longer than cells treated with paclitaxel and hesperadin, this indicates that the levels of Mad2 localization at kinetochores might be sufficient to sustain eribulin-induced mitotic arrest for some time after inhibition of Aurora B by hesperadin.

Aurora A is another member of the Aurora kinase family. TBA treatment induced not only activating phosphorylation of Aurora B at Thr-232, but also of Aurora A at Thr-288 during mitosis (Fig. 1B). A previous report showed that inhibition of Aurora A using the specific inhibitor MLN8054 or depletion of Aurora A via RNAi induces slippage from mitotic arrest induced by paclitaxel. In these conditions, Aurora B activity does not seem to be abrogated because the number of cells expressing histone H3 phosphorylated at Ser10 is not markedly decreased^37^. On the other hand, in this study, specific inhibition of Aurora B using barasertib did not prevent the activating phosphorylation of Aurora A at Thr-288 during slippage from mitotic arrest induced by paclitaxel. Hence, inhibition of Aurora A and Aurora B may induce slippage from paclitaxel-induced mitotic arrest via different mechanisms.

The cell fates following mitotic slippage distinctly differ between the types of slippage. In the case of paclitaxel, inhibition of Aurora B by hesperadin appears to play a direct role in preventing sensitivity to paclitaxel. One possible explanation is that inhibition of Aurora B rapidly leads to mitotic slippage in paclitaxel-treated cells; therefore, apoptotic signals do not reach the threshold required for cell death, resulting in survival after tetraploidy generation^15,38^. In contrast, cells treated with paclitaxel alone exit after prolonged mitotic arrest, and then die in the following interphase^15,38^. A previous study demonstrated that the main subpopulation of HeLa cells treated with nocodazole alone was arrested in mitosis and also exited mitosis but subsequently underwent endoreduplication, entering the next mitosis cycle without ever undergoing division^15^. Here, eribulin-treated cells might behave similar to nocodazole-treated cells. Importantly, in the case of eribulin, inhibition of Aurora B by hesperadin appeared to lead to enhanced sensitivity to eribulin (Fig. 4B–D,F). In our study, the duration of mitosis for cells treated with eribulin alone was longer than that for cells treated with eribulin and hesperadin (Fig. 2C); thus, the cytotoxicity of eribulin might not simply be associated with the duration of sustained mitosis. The contribution of SAC proteins, including Aurora B, to paclitaxel sensitivity has been previously reported in esophageal squamous cell carcinoma and gastric, ovarian, and breast cancer cells^39–42^. Furthermore, reduced expression of SAC components has been observed in several kinds of cancer cells, resulting in impaired ability to sustain SAC signaling^43^. Taken together with our results showing that inhibition of Aurora B leads to enhanced sensitivity to eribulin, this indicates that MT-destabilizing agents, such as vinblastine, vincristine, vinorelbine, and eribulin, may be more appropriate than MT-stabilizing agents, such as taxans and epothilones, for treating cancer cells with a weakened SAC. The difference between the roles played by inhibition of Aurora B in paclitaxel- and eribulin-treated cells is not yet clear. Further investigation of the molecular mechanisms underlying the contribution of inhibition of Aurora B in paclitaxel- and eribulin-treated cells will be required for understanding the effect of MT-stabilizing and MT-destabilizing agents–induced mitotic slippage on postmitotic cell fates.

Consistent with previous studies on therapy-induced senescence (TIS), including the inhibition of Aurora B, cellular senescence-like phenotypes were observed in HeLa cells with endoreduplication induced on treatment with paclitaxel and hesperadin and on treatment with eribulin and hesperadin. Although the causes and phenotypes of polyploid cells differed between these two types of treatments in the short term, the fate of cells, which underwent substantial endoreduplication and became highly polyploid, were similar under both conditions. Furthermore, in light of recent studies showing that various agents can induce cellular senescence^34^, it is suggested that the inhibition of Aurora B or other SAC components when tumor cells exhibit aberrant mitosis on treatment with TBAs can aid in tumor suppression in the form of a trigger for cellular senescence.

## Materials and Methods

### Cell culture, RNAi, and reagents

HeLa (obtained from JCRB Cell Bank) and MDA-MB-231 (obtained from ATCC) cells were cultured in Dulbecco’s modified Eagle’s medium (DMEM, Gibco). SK-BR-3 (obtained from ATCC) cells were cultured in McCoy’s 5 A medium (Gibco). T47D (ATCC) cells were cultured in RPMI 1640 medium (Gibco). HeLa.S-Fucci2 cells were provided by the RIKEN BRC through the National Bio-Resource Project of the MEXT, Japan. The cell lines were authenticated by short tandem repeat (STR) profiling and tested for mycoplasma contamination. HeLa cells stably expressing green fluorescent protein (GFP)–tagged histone H2B (provided by Dr. Hiroshi Kimura, Tokyo Institute of Technology, Japan) and HeLa.S-Fucci2 cells were used for live-cell imaging. For cell synchronization, HeLa cells were incubated in thymidine-containing medium for 18 h and released into fresh medium. At 6 h after release, the medium was replaced with a new one containing paclitaxel or eribulin. All media were supplemented with 10% fetal bovine serum, penicillin (100 U ml^−1^), and streptomycin (100 µg ml^−1^). All cells were grown in a 5% CO_2_ atmosphere at 37 °C. Aurora B-specific siRNA#1 (5′-AACGCGGCACUUCACAAUUGAdTdT-3′^21^) was synthesized by Takara Bio. Another siRNA#2 were ON-TARGET plus SMARTpool siRNAs targeting Aurora B (5′-CAGAAGAGCUGCACAUUUGdTdT-3′, 5′-GCGCAGAGAGAUCGAAAUCdTdT-3′,5′-ACGCGGCACUUCACAAUUGdTdT-3′, 5′-CCAAACUGCUCAGGCAUAAdTdT-3′), and were synthesized by Thermo Fisher. In all experiments, a duplex targeting the luciferase gene (GL3; 5′- CUUACGCUGAGUACUUCGAdTdT-3′) was used as a control. siRNA transfections were performed using Lipofectamine RNAiMAX (Thermo Fisher). The final concentration of each reagent was as follows: paclitaxel, 10 µM (T1912; Sigma); eribulin, 10–100 nM (Eisai Co. Ltd.); hesperadin, 50 nM (375680; Calbiochem); ZM447439, 2 µM (2458; Tocris Bioscience); barasertib (AZD1152-HQPA), 1 µM (S1147; Selleckchem); and thymidine, 2.5 mM (T1895; Sigma).

### Immunoblotting

Immunoblotting analyses were performed as previously described^44^. Briefly, cells were harvested and lysed in lysis buffer (20 mM Tris, pH 8.0, 150 mM NaCl, 1 mM EDTA, 0.5% NP-40, 1 mM phenylmethylsulfonyl fluoride, protease-inhibitor cocktail, and phosphatase-inhibitor cocktail [Nacalai Tesque]) for 30 min on ice. Cell extracts were clarified by centrifugation. Cell lysates were boiled in SDS loading buffer. Pellet fractions were analyzed as a chromatin-rich fraction. Western blotting of protein extracts was performed with standard methods, using the following antibodies: rabbit anti-Aurora B (1:1,000; 3094, Cell Signaling Technology), rabbit anti-phospho-Aurora A (T288)/phospho-Aurora B (T232)/phospho-Aurora C (T198) (1:2,000; 2914, Cell Signaling Technology); mouse anti-cyclin B1 (1:1,000; 05-373SP, Millipore); rabbit anti-phospho-histone H3 (Ser10) (1:1,000; 9071, Cell Signaling Technology); mouse anti-histone H3 (1:1,000; 3638, Cell Signaling Technology); mouse anti-phospho-Ser/Thr-Pro MPM-2 (1:500; 05-368, Millipore); rabbit anti-PARP (1:1,000; 9542, Cell Signaling Technology); rabbit anti-cleaved PARP (Asp214) (1:1,000; 5625, Cell Signaling Technology); mouse anti-caspase-9 (1:1,000; 9508, Cell Signaling Technology); rabbit anti-cleaved caspase-9 (1:1,000; 7237, Cell Signaling Technology); rabbit anti-p53 (1:500; sc-6243, Santa Cruz Biotechnology); rabbit anti-p21 (1:500; sc-397, Santa Cruz Biotechnology); mouse anti-p16 (1:100; sc-56330, Santa Cruz Biotechnology); and mouse anti-β-actin (1:5,000; A5316, Sigma) antibodies. Quantitative analysis was performed using the ImageJ software^45^.

### Immunofluorescence

Cells were rinsed in PBS at 37 °C, fixed in 4% paraformaldehyde for 15 min at 37 °C, permeabilized (PBS containing 0.1% Triton X-100) for 5 min, blocked (PBS containing 2% bovine serum albumin and 2% normal goat serum) for 30 min, and incubated with the following antibodies at the indicated dilution: mouse anti-α-tubulin (1:2,000; T6199; Sigma) and mouse anti-lamin A/C (1:200; 4777, Cell Signaling Technology). Secondary antibodies conjugated to Alexa Fluor 488 and 568 (Molecular Probes) were used at 1:2,000 dilution. After a 5-min wash in PBS containing 4′,6-diamidino-2-phenylindole (DAPI), the coverslips were mounted in ProLong Diamond (Thermo Fisher).

### Image acquisition and analysis

Image acquisition and analysis were performed as previously described^44^. Briefly, for fixed-cell experiments, fluorescence image acquisition was performed using a Nikon A1R confocal imaging system controlled by the Nikon NIS Elements software (Nikon). The objective lens was an oil immersion Plan-Apo 60× NA 1.40 lens (Nikon). Images were acquired as Z-stacks at 0.2-μm intervals, and maximum-intensity projections were generated using the NIS Elements software (Nikon). For live-cell imaging of mitotic duration and cell cycle progression, HeLa H2B-GFP and HeLa Fucci cells were imaged in 35-mm glass bottom dishes (Iwaki) containing FluoroBrite DMEM (Gibco). The imaging medium was maintained at 37 °C under 5% CO_2_ in a stage-top incubator (Tokai Hit). Images were acquired every 5 min for 24 h with a 20 ms exposure time by using a Plan-Apo 20× NA 0.75 lens (Nikon) objective on an inverted fluorescence microscope (Nikon Eclipse Ti-E) equipped with an ORCA-Flash 4.0 V2 camera (Hamamatsu Photonics). Mitotic duration was calculated manually.

### Fluorescence-activated cell sorting (FACS) analysis

For measurement of the mitotic index, cells were collected and fixed with ice-cold 70% ethanol for 1 h, treated with a blocking solution (PBS containing 5% fetal bovine serum) for 1 h, and then incubated with mouse anti-phospho-Ser/Thr-Pro MPM-2 (1:500; 05-368, Millipore) in the blocking solution for 1 h at 37 °C. This was followed by incubation for 1 h at room temperature with secondary antibodies conjugated to Alexa Fluor 488 (Molecular Probes). The cells were subsequently incubated with propidium iodide (PI) staining solution (5 μg/ml PI; 10 μg/ml Rnase A in PBS) for 30 min at 37 °C and analyzed using a FACS Calibur flow cytometer (BD Biosciences). The percentage of MPM-2 positive cells was determined in terms of mitotic cells.

### Cell viability and senescence assays

Cells were plated in 96-well plates at the following dilutions: HeLa cells at 5,000 cells/well; T47D and SK-BR-3 cells at 10,000 cells/well; and MDA-MB-231 cells at 7,500 cells/well. The cells were cultured in the continuous presence of test compounds for 3 days, except for HeLa cells, which were cultured in the presence of test compounds after synchronization of mitosis by a single thymidine block for 18 h. Cell viability and senescence-associated expression of β-galactosidase (SA-β-Gal) were assessed using the CellTox Green Cytotoxicity Assay (Promega) and the Senescence Detection kit (BioVision), respectively, according to the manufacturers’ instructions.

### Statistical analysis

The data were analyzed using Student’s *t*-test or the Mann–Whitney *U*-test. We tested the data for normality and variance between groups. Statistical calculations were performed using the JMP program (SAS institute Inc). All immunoblotting and immunofluorescence experiments were repeated twice.

## Electronic supplementary material

Supplementary Information
