# Supplementary material for: Mitotic slippage and the subsequent cell fates after inhibition of Aurora B during tubulin-binding agent–induced mitotic arrest
Source: Sci Rep. 2017 Dec 1;7:16762. doi: 10.1038/s41598-017-17002-z (PMC5711930; doi:10.1038/s41598-017-17002-z)
Supplement: Supplementary file 1 — Supplementary Information [file 41598_2017_17002_MOESM1_ESM.pdf]

Supplementary information for

Mitotic slippage and the subsequent cell fates after inhibition of Aurora B during tubulin-binding agent–induced mitotic arrest

Yasuo Tsuda[1], Makoto Iimori[2]*, Yuichiro Nakashima[1], Ryota Nakanishi[1], Koji Ando[1], Kippei Ogaki[1], Hiroyuki Kitao[2], Hiroshi Saeki[1], Eiji Oki[1], and Yoshihiko Maehara[1]

1: Departments of Surgery and Science, Graduate School of Medical Sciences, Kyushu University, 3-1-1 Maidashi, Higashi-ku, Fukuoka, 812-8582 Japan

2: Department of Molecular Cancer Biology, Graduate school of Pharmaceutical Sciences, Kyushu University, 3-1-1 Maidashi, Higashi-ku, Fukuoka, 812-8582, Japan

*Corresponding author: Makoto Iimori

Phone/FAX: +81-92-642-6499; E-mail: iimori@surg2.med.kyushu-u.ac.jp

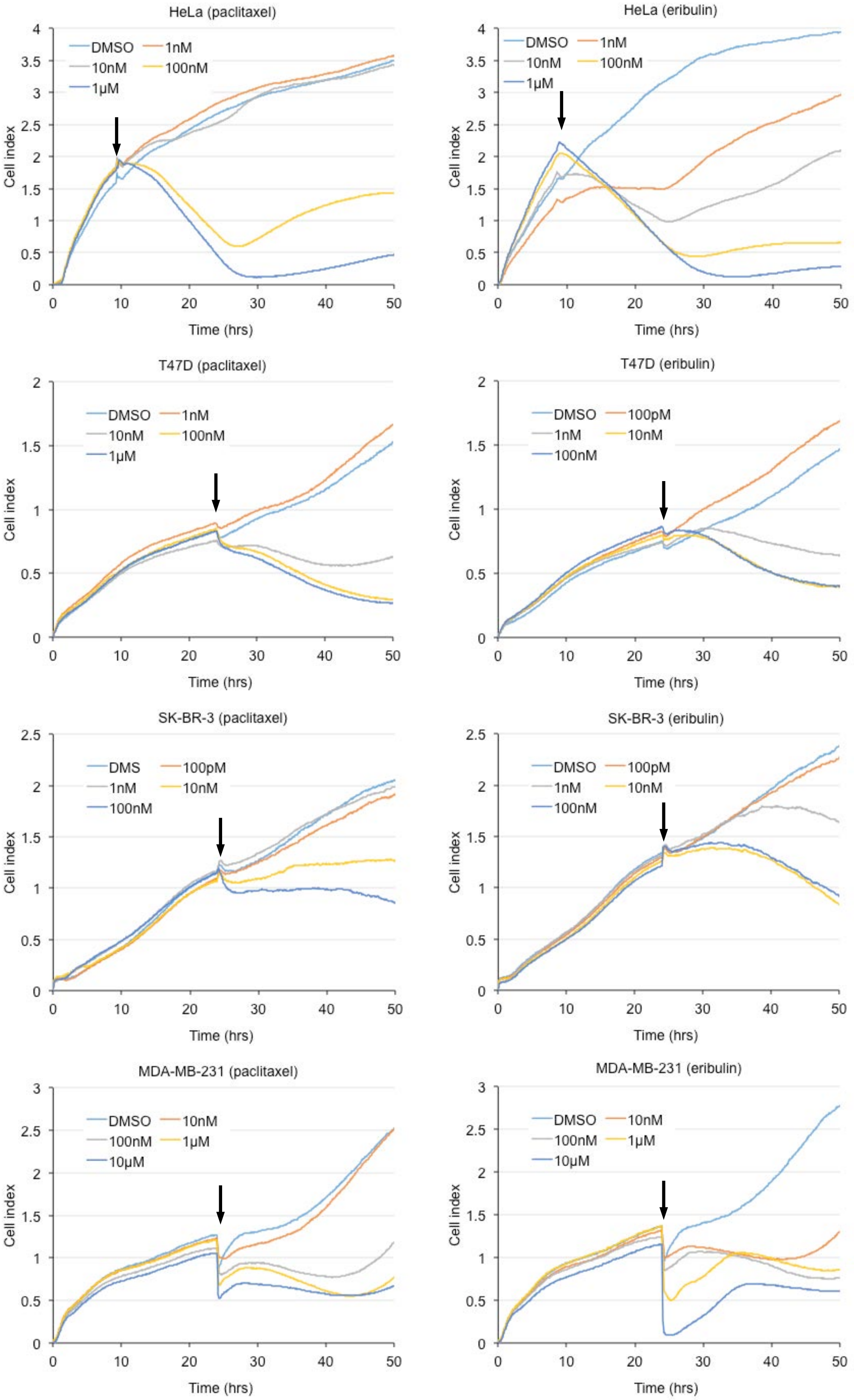

Supplementary figure 1. Optimization of minimum concentrations of paclitaxel and eribulin.

Cell index was continuously monitored with the xCELLigence technology, which is a real-time cellular biosensor. Arrows indicate the time points of paclitaxel and eribulin treatment.

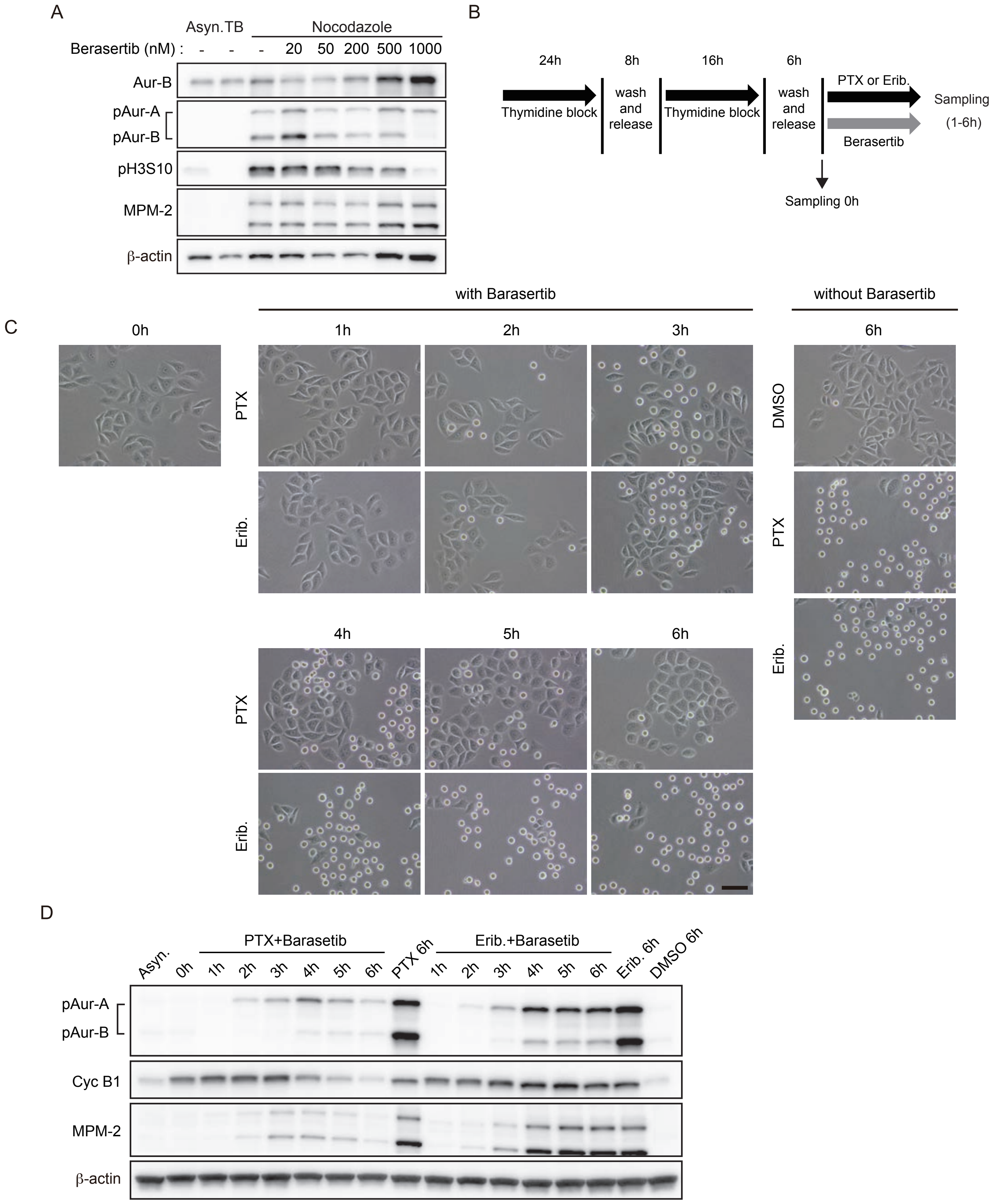

Supplementary figure 2. Effect of inhibition of Aurora B by using barasertib during paclitaxel- or eribulin-induced mitotic arrest in HeLa cells.

(A) Validation of the optimal concentration of barasertib for specific inhibition of Aurora B. Synchronized HeLa cells were treated with 5 points of dilution series of barasertib during mitotic arrest by nocodazole treatment. Immunoblot analysis was performed using antibodies against the indicated proteins. The levels of the mitosis-specific markers MPM-2 were determined. (B) Schemes of experiments shown in Supplementary Fig. 2C and D. (C) Representative images showing the morphology of cells in each sampling time. Scale bar, 100 μm. (D) Synchronized HeLa cells were treated with 100 nM PTX or 10 nM Erib with or without 1 μM barasertib. HeLa cells that underwent mitosis arrest were harvested in each sampling time. Immunoblot analysis was performed using antibodies against the indicated proteins. The levels of the mitosis-specific markers MPM-2 and cyclin B1 were determined.

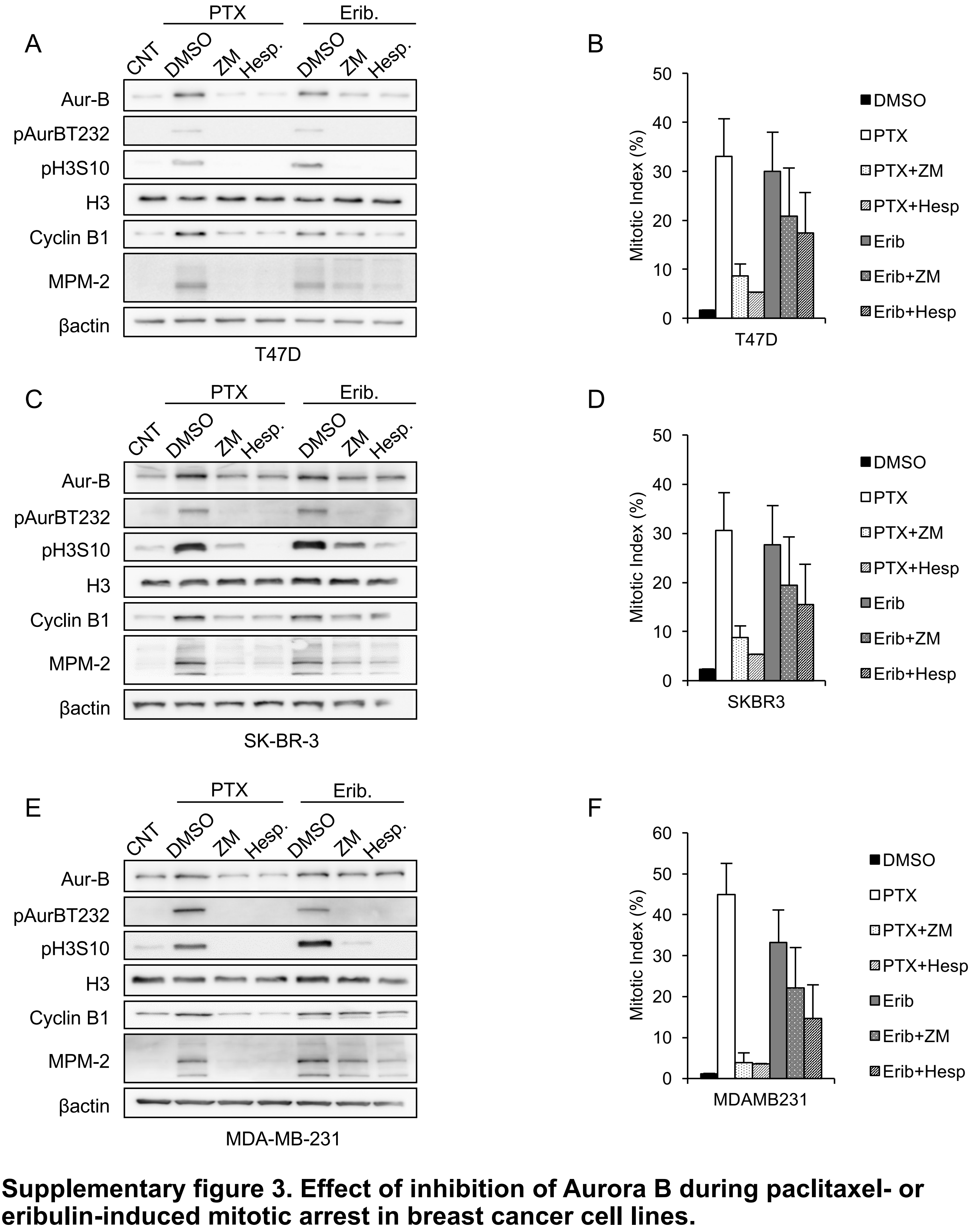

Supplementary figure 3. Effect of inhibition of Aurora B during paclitaxel- or eribulin-induced mitotic arrest in breast cancer cell lines.
(A, B: T47D; C, D: SK-BR-3; E, F: MDA-MB-231) Breast cancer cells were were cultured with 100 nM PTX or 10 nM Erib for 16 h. Cells that underwent mitosis arrest were transferred into fresh medium containing added DMSO, 2μM ZM447439 (ZM), or 50nM Hesperadin (Hesp). (A, C, E) Immunoblot analysis was performed using antibodies against the indicated proteins. The levels of the mitosis-specific markers MPM-2 and cyclin B1 were determined. (B, D, F) The mitotic index was determined by analyzing MPM2-positive cells. Data are mean values from three independent experiments ; error bars, ± SD, number of cells, >10,000 per experiment.

PTX+Hesp.

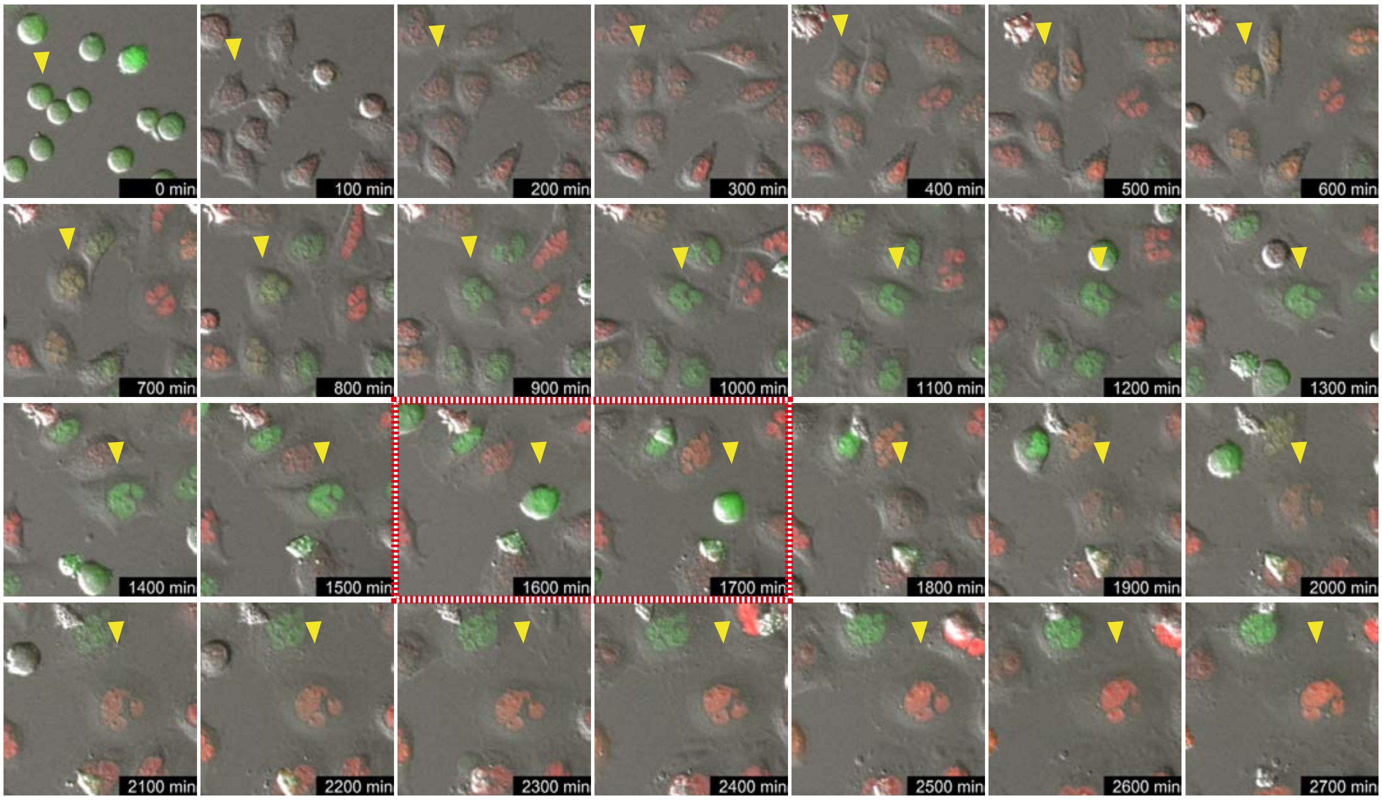

Erib.+Hesp.

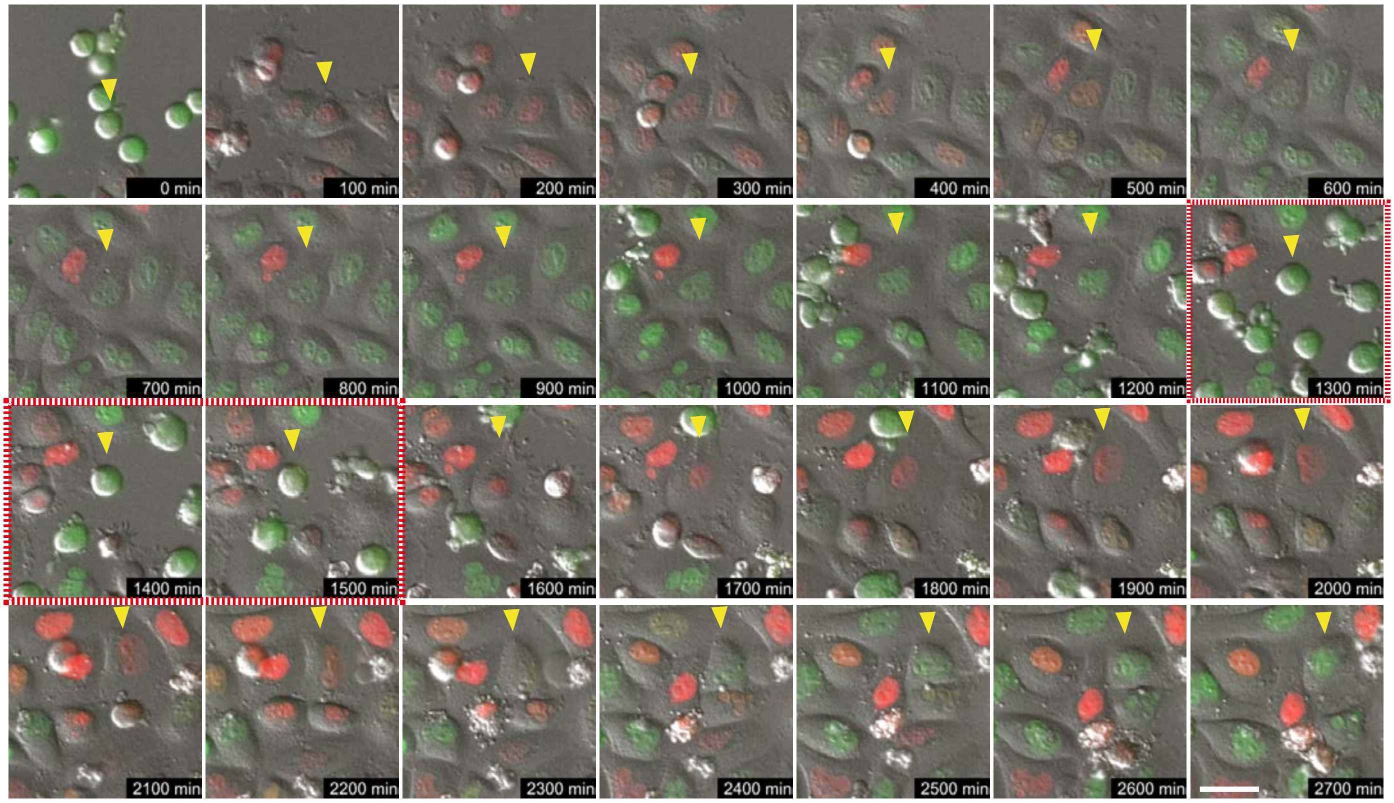

Supplementary figure 4. Cell cycle progression of inhibition of Aurora B during paclitaxel- or eribulin-induced mitotic arrest in HeLa-Fucci cells.
Select frames from live-cell imaging of HeLa-Fucci cells. Synchronized HeLa-Fucci cells were cultured with 100 nM paclitaxel (PTX) or 10 nM eribulin (Erib) for 6 h after release. Hesperadin (Hesp) was added to cells arrested in mitosis by treatment with PTX or Erib (0 min) . Representative cell images of post mitotic slippage are indicated by yellow arrowheads. Red boxes show the cells underwent re-entry to the next mitosis.

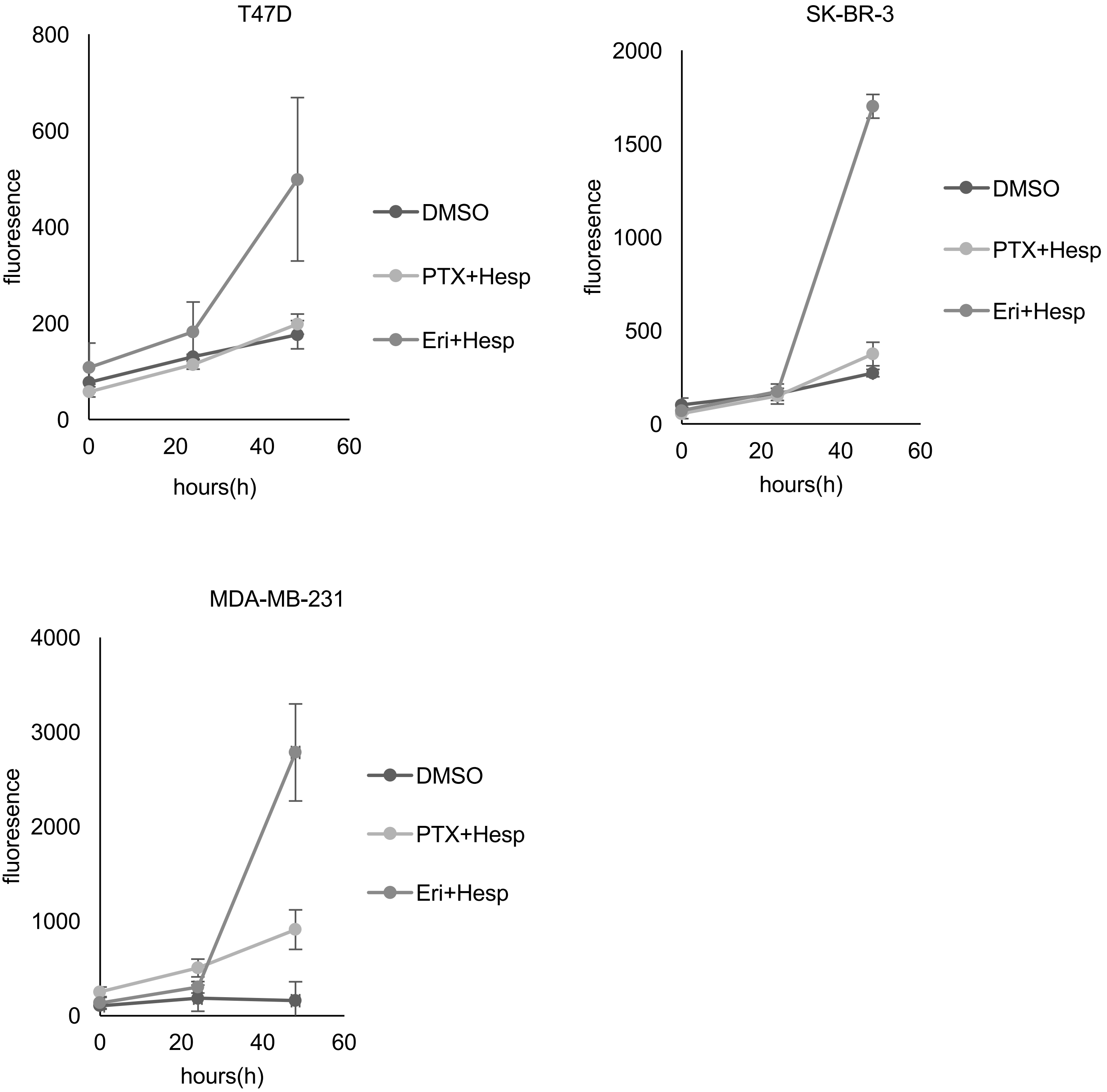

Supplementary figure 5. Cytotoxicity in postmitotic slippage in breast cancer cell lines.
Cell viability was analyzed using the CellTox Green Cytotoxicity Assay.
